# The Effect of Posterior Pericardiotomy on the Incidence of Atrial Fibrillation After Cardiac Surgery–Extended Follow-Up study (PALACS-EF): rationale and design

**DOI:** 10.1093/ehjopen/oead118

**Published:** 2023-11-17

**Authors:** Mario Gaudino, Lamia Harik, Bjorn Redfors, Sigrid Sandner, John H Alexander, Antonino Di Franco, Arnaldo Dimagli, Jonathon Weinsaft, Roberto Perezgrovas-Olaria, Giovanni Jr Soletti, Christopher Lau, Charles Mack, Leonard Girardi

**Affiliations:** Department of Cardiothoracic Surgery, Weill Cornell Medicine, 525 E 68th St, New York, NY 10065, USA; Department of Cardiothoracic Surgery, Weill Cornell Medicine, 525 E 68th St, New York, NY 10065, USA; Department of Cardiology, Sahlgrenska University Hospital, Gothenburg, Sweden; Department of Cardiac Surgery, Medical University of Vienna, Vienna, Austria; Division of Cardiology, Department of Medicine, Duke Clinical Research Institute, Duke Health, Durham, NC, USA; Department of Cardiothoracic Surgery, Weill Cornell Medicine, 525 E 68th St, New York, NY 10065, USA; Department of Cardiothoracic Surgery, Weill Cornell Medicine, 525 E 68th St, New York, NY 10065, USA; Department of Cardiothoracic Surgery, Weill Cornell Medicine, 525 E 68th St, New York, NY 10065, USA; Department of Cardiothoracic Surgery, Weill Cornell Medicine, 525 E 68th St, New York, NY 10065, USA; Department of Cardiothoracic Surgery, Weill Cornell Medicine, 525 E 68th St, New York, NY 10065, USA; Department of Cardiothoracic Surgery, Weill Cornell Medicine, 525 E 68th St, New York, NY 10065, USA; Department of Cardiothoracic Surgery, Weill Cornell Medicine, 525 E 68th St, New York, NY 10065, USA; Department of Cardiothoracic Surgery, Weill Cornell Medicine, 525 E 68th St, New York, NY 10065, USA

**Keywords:** Cardiac surgery, Postoperative atrial fibrillation, Posterior pericardiotomy

## Abstract

**Aims:**

Postoperative atrial fibrillation (POAF) is the most common complication of cardiac surgery and has been associated with increased postoperative morbidity and hospital costs. The Posterior left pericardiotomy for the prevention of AtriaL fibrillation After Cardiac Surgery (PALACS) trial found that posterior pericardiotomy significantly reduced the incidence of POAF (17% vs. 32%, *P* < 0.001). We present the protocol for The Effect of Posterior Pericardiotomy on the Incidence of Atrial Fibrillation After Cardiac Surgery–Extended Follow-Up study (PALACS-EF): a prospective, extended follow-up of the original PALACS trial. The aim of PALACS-EF is to gain more data regarding the effect of posterior pericardiotomy on postdischarge clinical outcomes. The primary outcome is the time to the first occurrence of the composite of all-cause mortality or hospital cardiovascular readmission. The key secondary outcome is the time to the first occurrence of the composite of all-cause mortality and all-cause hospital readmission. Hospital readmission, myocardial infarction, stroke, transient ischaemic attack, heart failure, systemic embolism, or new arrhythmias with onset since 30-day follow-up will also be recorded.

**Methods and results:**

All 420 patients enrolled in the PALACS trial will be included; extended follow-up will be conducted via telephone by blinded research personnel utilizing a standardized script to ensure uniformity and completeness of follow-up. If an event has occurred, documentation will be obtained, and an independent adjudication committee blinded to group assignment will adjudicate outcome events. Results will be reported when a median follow-up of 5 years is achieved.

**Conclusion:**

PALACS-EF will provide data to answer the question of whether posterior pericardiotomy improves postdischarge outcomes in patients undergoing cardiac surgery, and it will provide information on the relationship between POAF and adverse postdischarge outcomes including mortality, hospitalization, heart failure, and stroke.

**Registration:**

PALACS: NCT02875405, PALACS-EF: NCT05903222

## Graphical Abstract

**Graphical Abstract:**
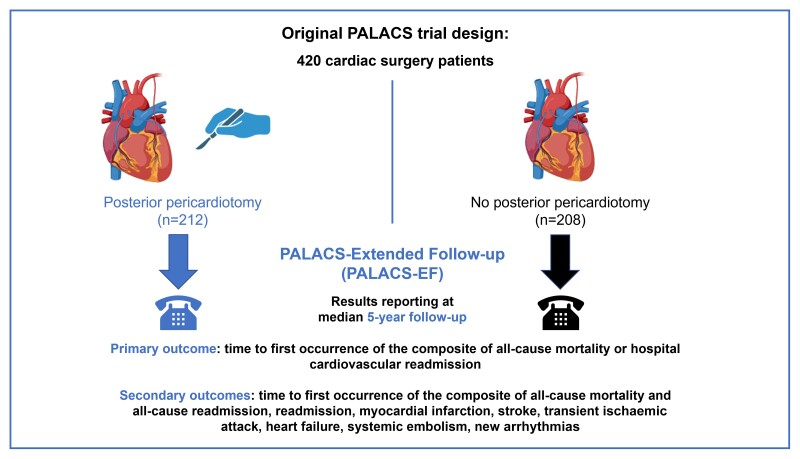

## Introduction

Postoperative atrial fibrillation (POAF) is the most common complication after cardiac surgery,^1–3^ and the most frequent type of secondary atrial fibrillation,^4^ with an incidence ranging from 19 to 30% in different series.^1,4,5^ Postoperative atrial fibrillation has been associated with increased mortality, higher rates of stroke, and heart failure,^2^ as well as with prolonged hospitalization and increased hospital costs,^1^ with an estimated annual healthcare expenditure in the USA exceeding 2 billion dollars.^6^ Despite a strong association, it is still unclear whether these outcome events are causally related to POAF or a simple association.^7–9^ Postoperative atrial fibrillation is also significantly associated with a higher risk of recurrent atrial fibrillation in the years after surgery;^10,11^ while it seems possible that the reported increased late risk of stroke, heart failure, and mortality associated with POAF might be mediated by clinical atrial fibrillation (i.e. atrial fibrillation occurring outside the postoperative period), to date, this has not been clearly proven,^7,8^ and it is not known at present if strategies that reduce the risk of POAF also improve clinical outcomes in the years after surgery.

Numerous strategies aimed at reducing POAF incidence have been investigated, including pharmacological and non-pharmacological interventions, all with limited results and potentially relevant side effects.^12^ The efficacy of left posterior pericardiotomy, a surgical procedure that allows drainage of the pericardium into the left pleural space, has been initially tested in several small randomized clinical trials (RCTs).^7^ Recently, the Posterior left pericardiotomy for the prevention of AtriaL fibrillation After Cardiac Surgery (PALACS) trial^13^ confirmed on a larger scale (420 patients) that the intervention largely and significantly reduced the incidence of POAF by >50% [17% vs. 32%; *P* < 0.001; odds ratio (OR) 0.44, 95% confidence interval (CI) 0.27–0.70; *P* < 0.001].

The aim of The Effect of Posterior Pericardiotomy on the Incidence of Atrial Fibrillation After Cardiac Surgery–Extended Follow-Up study (PALACS-EF) is to gain more data regarding postdischarge outcomes of cardiac surgical patients who have received posterior pericardiotomy.

The results of this study will also contribute to the understanding of the nature of the POAF relationship with postoperative clinical outcomes.

## Methods

### Objective

The primary aim of the PALACS trial was to assess whether performing a posterior left pericardiotomy during open cardiac surgery procedures results in a reduction in the incidence of POAF.

The aim of PALACS-EF study is to gain more data regarding the effect of posterior pericardiotomy on postdischarge outcomes including all-cause mortality, hospital readmission for cardiovascular causes, all-cause hospital readmission, myocardial infarction (MI), stroke, transient ischaemic attack (TIA), heart failure, systemic embolism, or new arrhythmias with onset since 30-day follow-up. Institutional Review Board approval for the PALACS-EF protocol was given by Weill Cornell Medicine (IRB #1502015867) as an amendment to the original PALACS trial protocol approval, without the need for updated informed consent. No new data were generated or analysed for the present manuscript.

### Methods

Extended follow-up will be conducted via telephone by research personnel blinded to group assignment in the 420 patients enrolled in the initial PALACS trial. A standardized script will be used during telephone follow-up to ensure uniformity and completeness of follow-up (Appendix). If an event has occurred, documentation will be obtained. Initial patient contact for extended follow-up will occur in the order of patient enrolment. Patients will be contacted every 6 months, with time zero being the time of randomization in the PALACS trial.

An independent committee made of two cardiologists and a cardiac surgeon blinded to group assignment will adjudicate all outcome events. A neurologist blinded to group assignment will confirm all neurologic events, including TIA and stroke.

### Data management

All data will be de-identified upon entry into the secure, password-protected, electronic data collection system, REDCap. Data will be prospectively entered by research personnel. REDCap will be hosted on a secure server on a password-protected computer, and access to the database will require a password as well as permission from the principal investigator. The secure server being used is also password-protected and only accessible to research personnel of the Department of Cardiothoracic Surgery, Weill Cornell Medicine.

### Outcomes

The primary outcome of PALACS-EF is the time to the first occurrence of the composite of all-cause mortality or hospital readmission for cardiovascular causes.

The key secondary outcome is the time to the first occurrence of the composite of all-cause mortality or all-cause hospital readmission.

Additional secondary outcomes include all-cause hospital readmission, hospital readmission for cardiovascular causes, heart failure, MI, stroke, TIA, new arrhythmias with onset after 30-day follow-up, and systemic embolism. All-cause hospital readmission and hospital readmission for cardiovascular causes will be analysed both as time to first occurrence and as recurrent events.

Definitions for the study outcomes are provided in *Table 1*.

**Table 1:** Outcome definitions

Outcome	Definition
Heart failure^14^	Symptoms and/or signs of heart failure, including but not limited to:
	dyspnoea, orthopnoea, paroxysmal nocturnal dyspnoea
	reduced exercise tolerance or fatigue
	pedal or peripheral oedema, unintentional weight gain, elevated jugular venous pressure, new cardiac murmur or gallop, laterally displaced apical impulse, hepatojugular reflux
	Caused by a structural and/or functional cardiac abnormality, as determined by:
	an EF <50%
	abnormal cardiac chamber enlargement
	*E*/*e*′ of >15 interpreted as elevated left ventricular end-diastolic pressure
	moderate/severe ventricular hypertrophy
	moderate/severe valvular obstructive or regurgitant lesion
	Corroborated by one of the following:
	elevated natriuretic peptide levels (BNP ≥100 pg/mL at hospitalization or NT-proBNP ≥300 pg/mL at hospitalization)
	objective evidence of cardiogenic, pulmonary, or systemic congestion (chest X-ray, elevated filling pressures on echocardiography, haemodynamic measurement at rest or with provocation)
Myocardial infarction	The Fourth Universal Definition^15^:
	Perioperative myocardial infarction: cTn values >10 × 99th percentile of the URL during the first 48 h following surgery, occurring from a normal baseline cTn value (≤99th percentile URL). In addition, any of the following:
	new pathological Q-waves or new LBBB
	angiographically documented new graft or new native coronary artery occlusion
	imaging evidence of new loss of viable myocardium or new regional wall motion abnormality
	Non-perioperative myocardial infarction: detection of rise and/or fall of cardiac biomarkers with at least one value above the 99th percentile of the URL together with evidence of myocardial ischaemia with at least one of the following:
	symptoms of ischaemia
	ECG changes indicative of new ischaemia (new ST-T changes or new LBBB)
	development of pathological Q-waves in the ECG
	imaging evidence of new loss of viable myocardium or new regional wall motion abnormality
New arrhythmia	Newly diagnosed or new onset of any abnormal heart rhythm in the time since 30-day follow-up after surgery, confirmed by clinical diagnosis. Events will be categorized as atrial fibrillation or non-atrial fibrillation.
Stroke	Any neurological deficit of abrupt onset caused by a disturbance in blood supply to the brain that was confirmed on imaging or did not resolve within 24 h
Systemic embolism	Systemic embolism will be defined as acute vascular occlusion of an extremity or organ, confirmed by computed tomography or angiography
Transient ischaemic attack	A transient episode of neurologic dysfunction caused by focal brain, spinal cord, or retinal ischaemia, without acute infarction, with symptoms lasting <24 h

BNP, B-type natriuretic peptide; cTn, cardiac troponin; ECG, electrocardiogram; EF, ejection fraction; LBBB, left bundle branch abnormality; URL, upper reference limit.

### Inclusion criteria

The 420 patients enrolled in the primary PALACS trial will be part of PALACS-EF study. In PALACS, three patients (0.7%) died before hospital discharge or within 30 days of surgery. Those deaths will be counted towards the primary endpoint in the PALACS-EF study, but these patients will be excluded from the secondary analysis comparing the outcomes of patients with vs. without POAF. Inclusion criteria for the initial trial were as follows: patients undergoing primary, elective coronary artery bypass grafting, aortic valve replacement, ascending aortic repair, or a combination of these, who had no previous history of atrial fibrillation or other atrial arrhythmias.

### Exclusion criteria

The exclusion criteria for the primary PALACS trial were as follows: pre-operative non-sinus rhythm, history of previous atrial arrhythmias, mitral or tricuspid valve surgery, surgery of the descending thoracic or thoraco-abdominal aorta, planned use of hypothermic circulatory arrest at surgery, off-pump operation, urgent or emergent presentation, disease of the left pleura or previous interventions in the left pleural cavity, non–cardiac-related contraindications to the intervention, repeat operation, minimally invasive operation, and chest deformity.

### Statistical analysis plan

For this reporting, the primary analysis will be performed at a median follow-up of 5 years.

The primary analysis will compare the primary outcome by randomized treatment according to the intention-to-treat (ITT) principle where all participants will be included in their assigned treatment groups regardless of the actual procedure performed. The primary analysis will be performed using multivariable-adjusted Cox proportional hazards regression, adjusted for the following covariates: age, sex, diabetes status, ejection fraction, extent of coronary disease, type of surgery, and surgical priority. An as-treated (AT) analysis will also be conducted.

Key secondary analyses include (i) a comparison of the primary endpoint for patients with POAF vs. patients without POAF and (ii) a mediation analysis using randomized treatment as exposure, POAF as the mediator, and the primary outcome as the outcome. Both key secondary analyses will be performed in the ITT population using multivariable-adjusted Cox proportional hazards regression. Other secondary analyses will be performed both in the ITT and AT populations.

For the primary outcome, subgroup and interaction-term analyses will be used to investigate the following possible effect modifiers: age, sex, left ventricular ejection fraction, CHA_2_DS_2_-VASc score, and type of surgery.

The authors expect only minimal missing data. In case of missing data, multiple imputation analysis will be utilized. The loss to follow-up is also expected to be minimal. It will be reported and compared between the groups using absolute risk differences. All *P* values will be two sided.

Secondary outcomes will be analysed using Cox proportional hazards regression (time to first occurrence) and by the Lei Wei Yang Ying adaptation of the Anderson–Gil model with robust standard errors.^16^

Pre-operative differences between groups will be assessed by univariate analysis. Continuous variables will be analysed by way of Student’s *t*-test or the Mann–Whitney *U* test, and categorical variables will be analysed by use of the *χ*^2^ test.

### Statistical power

No formal sample size calculation has been performed for PALACS-EF. The 420 patients enrolled in the PALACS trial will be included in the extended follow-up study.

Based on the enrolled sample size, statistical power in different scenarios of event rates and treatment effects are provided in *Table 2*.

**Table 2:** Power estimates in different scenarios of event rates and treatment effects

5-year event rate (pooled)	Power assuming *n* = 420		
	HR = 0.70	HR = 0.65	HR = 0.60
50.0%	73.4%	87.7%	95.9%
47.5%	71.3%	86.1%	95.1%
45.0%	68.9%	84.2%	94.0%
42.5%	66.5%	82.2%	92.7%
40.0%	63.7%	80.0%	91.2%
37.5%	61.1%	77.3%	89.4%
35.0%	58.0%	74.3%	87.2%
32.5%	55.1%	71.2%	84.8%
30.0%	51.7%	67.6%	81.8%

HR, hazard ratio.

## Discussion

The PALACS trial was the first adequately powered, rigorous, randomized trial to evaluate the use of posterior pericardiotomy for prevention of POAF. This single-centre, single-blind, prospective trial found that compared with the control group, patients receiving posterior pericardiotomy had significantly lower incidence of POAF [17% (37/212) vs. 32% (66/208), *P* = 0.0007; OR adjusted for the stratification variable 0.44, 95% CI 0.27–0.70, *P* = 0.0005] and pericardial effusion [12% (26/209) vs. 21% (45/211); relative risk 0.58, 95% CI 0.37–0.91]. There was no difference between groups in the incidence of short-term major adverse events, and there were no pericardiotomy-related complications. A 2023 meta-analysis^17^ including 25 RCTs comparing posterior pericardiotomy to no posterior pericardiotomy in 4467 patients undergoing cardiac surgery found that the overall risk of POAF was significantly lower in the posterior pericardiotomy group (OR 0.49, 95% CI 0.38–0.610), as was the risk of supraventricular tachycardia (0.66, 95% CI 0.43–0.89), early (0.32, 95% CI 0.22–0.46) and late pericardial effusion (OR 0.15, 95% CI 0.09–0.25), and cardiac tamponade (OR 0.18, 95% CI 0.09–0.25). No data on mid- or long-term clinical outcomes were reported.

To date, it is not clear if posterior pericardiotomy improves postdischarge outcomes in patients undergoing cardiac surgery, and PALACS-EF will provide data on this important issue. In addition, PALACS-EF will provide information on the relationship between POAF and adverse postdischarge outcomes including mortality, hospitalization, heart failure, and stroke.

## Data Availability

No new data were reported, generated, or analysed for the present manuscript.

## Lead author biography

Dr Mario Gaudino is the Stephen and Suzanne Weiss Professor in Cardiothoracic Surgery at New York Presbyterian/Weill Cornell Medicine. He is an expert in coronary artery bypass surgery and the lead investigator of international trials aiming to determine the optimal strategy for coronary artery bypass surgery (ROMA and ROMA:Women). He is the Director of Translational and Clinical Research in the Department of Cardiothoracic Surgery and the Director for the Joint Clinical Trials Office at Weill Cornell Medicine/New York-Presbyterian, where he oversees clinical trials and provides strategic growth plans to increase investigator-initiated and pragmatic trials and to enhance clinical trial infrastructure.

## Appendix

### Script for PALACS trial follow-up

Good morning/afternoon Mr./Mrs. *last name*. I am Dr. *last name*, one of the Research Fellows working in the Department of Cardiac Surgery at Weill Cornell.

I am reaching out to ask you a couple of questions related to you participation in the PALACS trial.

1. Have you been READMITTED TO THE HOSPITAL SINCE YOUR SURGERY?
  1.1 No (go to question 2)
  1.2 Yes:
    - **When** were you readmitted?
    - What was the **reason for readmission**?
    - Ask patient to send documentation
2. Have you had **ANY OTHER HEALTH ISSUE SINCE YOUR SURGERY** (even if it did not lead to readmission)?
  2.1 No (go to question 3)
  2.2 Yes:
    - **What health issue or issues?**
      - **Date**
3. Have you been diagnosed with ATRIAL FIBRILLATION or ANY OTHER ARRHYTHMIA since discharge?
  3.1 No (go to question 4)
  3.2 Yes:
    - **When** were you diagnosed?
    - What was the **clinical presentation of the arrhythmia**?
      - Asymptomatic
      - Symptomatic but stable
      - Hemodynamic instability
    - Did the arrhythmia require treatment at the time of presentation?
      - No treatment
      - Pharmacological treatment
      - Cardioversion
4. Are you currently taking any **MEDICATIONS**?
  4.1 No (go to final line)
  4.2 Yes:
    - **Any of the following?**
      - Warfarin (coumadin, jantoven)
      - Apixaban (eliquis), Rivaroxaban (Xarelto)
      - Beta blockers
      - Amiodarone
      - Other (please indicate) (space available for free-text entry)

*Ask patient to provide email address*  **AND** contact of the referring cardiologist if not in the system.

Well, Mr./Mrs. *last name* those are all the questions we had for you today. **We’ll call you again 6 months from now** just to check on your health status.

Thank you for taking the time.
